# Meta-analysis of Urine Heme Dipstick Diagnosis of *Schistosoma haematobium* Infection, Including Low-Prevalence and Previously-Treated Populations

**DOI:** 10.1371/journal.pntd.0002431

**Published:** 2013-09-12

**Authors:** Charles H. King, David Bertsch

**Affiliations:** 1 Center for Global Health and Diseases, Case Western Reserve University School of Medicine, Cleveland, Ohio, United States of America; 2 Schistosomiasis Consortium for Operational Research and Evaluation, University of Georgia, Athens, Georgia, United States of America; University of Nottingham, United Kingdom

## Abstract

**Background:**

Urogenital schistosomiasis remains highly endemic in Africa. Current control is based on drug administration, targeted either to school-age children or to high-risk communities at-large. Urine dipsticks for detection of microhematuria offer an inexpensive means for estimating infection prevalence. However, their diagnostic performance has not been systematically evaluated after community treatment, or in areas with continuing low prevalence. The objective of the present study was to perform meta-analysis of dipstick accuracy for *S. haematobium* infection in endemic regions, with special attention to performance where infection intensity or prevalence was low.

**Methodology/Principal Findings:**

This review was registered at inception with PROSPERO (CRD42012002165). Included studies were identified by computerized search of online databases and hand search of bibliographies and existing study archives. Eligible studies included published or unpublished population surveys irrespective of date, location, or language that compared dipstick diagnosis of *S. haematobium* infection to standard egg-count parasitology. For 95 included surveys, variation in dipstick sensitivity and specificity were evaluated according to study size, age- and sex-specific participation, region, local prevalence, treatment status, and other factors potentially affecting test performance. Independent of prevalence, accuracy was greater in surveys of school-age children (*vs.* adults), whereas performance was less good in North Africa, as compared to other regions. By hierarchical ROC analysis, overall dipstick sensitivity and specificity for detection of egg-positive urine were estimated at 81% and 89%, respectively. Sensitivity was lower among treated populations (72%) and in population subgroups having lower intensity infection (65%). When the insensitivity of egg count testing was considered (and diagnosis inferred instead from combined hematuria and egg-count findings), overall dipstick sensitivity/specificity were 82%/97%, with significantly better sensitivity (92%) in high prevalence settings.

**Conclusions/Significance:**

This analysis suggests that dipsticks will continue to serve as very useful adjuncts for monitoring community prevalence following implementation of population-based control of urogenital schistosomiasis.

## Author Summary

Schistosomiasis is a chronic human disease caused by infection with multicellular trematode parasites of *Schistosoma* species. In particular, *Schistosoma haematobium* colonizes the veins in the pelvis, in and around the urinary tract, and causes inflammation that leads to ulceration and bleeding into the urine. One low-tech, inexpensive means of quickly identifying blood in the urine (hematuria) is to use a chemical reagent strip (dipstick) to test a patient's urine. While many studies have confirmed the usefulness of dipstick-detected hematuria as a proxy for infection in diagnosing infected people before treatment is given, their diagnostic performance after treatment has been uncertain. The current study systematically reviewed 95 available reports of dipstick performance in *S. haematobium-*endemic areas of Africa and determined, based on a meta-analysis of the primary data, that dipsticks appear to retain their diagnostic accuracy in low prevalence areas and after one or more rounds of treatment. This suggests that dipsticks will continue to be very useful tools in tracking and targeting regional requirements for treatment and prevention of *S. haematobium* infection as current school- and community-based programs go forward.

## Introduction

Urogenital schistosomiasis caused by *Schistosoma haematobium* infection is highly endemic in many developing areas of Africa and the Middle East that lack adequate sanitation and safe water supply [1]–[5]. The urinary dipstick for detection of hematuria has long been recommended as a relatively inexpensive and potentially accurate proxy for detection of *S. haematobium* infection [6]–[12], but as with any diagnostic test, performance characteristics can vary with the underlying population prevalence of the targeted disease [13], [14]. Multi-center trials by the Red Urine Study Group and others have documented the utility of using gross hematuria or a questionnaire-derived history of visible hematuria to identify schools or communities with a high prevalence of *S. haematobium* infection [15]–[19]. Current WHO guidelines for preventive chemotherapy recognize hematuria prevalence, in addition to egg count-based criteria, as an effective means for identifying communities with high, moderate, or low risk for schistosomiasis [20]. However, reports from programs performing repeated intervention for schistosomiasis indicate that visible (gross) hematuria dramatically declines following therapy, and so it can no longer be used as an indicator of local prevalence of urogenital schistosomiasis as campaigns proceed [21]. The question remains: does microscopic hematuria, detectable by dipstick, continue to be a good marker for *S. haematobium* infection after treatment? Whereas heme dipstick performance, *per se*, has been extensively examined in *S. haematobium-*endemic communities when tested before therapy [22], dipstick performance after mass treatment, or in communities with marginal transmission, has remained uncertain [23], [24].

In the present meta-analysis, we sought to assess systematically the diagnostic performance of urinary ‘chemical reagent dipsticks’ (those that detect urine heme) for the diagnosis of *S. haematobium* in both high- and low-prevalence areas, using available published and unpublished evidence. In particular we sought to estimate the utility of dipstick diagnosis for estimation of community prevalence in ongoing campaigns for schistosomiasis control. Our systematic review focused on results of studies that specifically compared dipstick hematuria to urine egg positivity at the individual subject level in population-based surveys for *S. haematobium* infection. The studies included were single or repeated cross-sectional surveys of either school-age or community-based groups in any endemic area, and our summary estimates of dipstick diagnostic performance were based on meta-analysis of data from 95 surveys identified in our systematic review.

## Methods

### Protocol and registration

The protocol for this project was developed prospectively by the authors then registered and published in the International Prospective Register of Systemic Reviews (PROSPERO) online database, http://www.crd.york.ac.uk/prospero/index.asp, number CRD42012002165, on 03 January 2012.

### Ethics statement

The data used in this project were aggregated, anonymized data from previously published studies; as such, this study does not constitute human subjects research according to U.S. Department of Health and Human Services guidelines (http://www.hhs.gov/ohrp/policy/checklists).

### Eligibility criteria

In assessing the diagnostic performance of chemical reagent strips (urine dipsticks) for the detection of hematuria as a proxy diagnosis for *S. haematobium* infection, we aimed to include any available published or unpublished school-age or community-based population surveys, irrespective of date, location, or language of report. Studies had to include paired data for comparison of both dipstick hematuria and egg output, at the per-subject level, in order to provide study-specific estimates for true positive/true negative/false positive/false negative categories. Hospital-based or case-series, in which representativeness of the sample to the general population was unknown, were excluded. We aimed to include any studies in English, Spanish, French, Arabic, Chinese, or Portuguese in which dipstick performance was quantitatively measured for the period of 1 Jan 1966 to 31 July 2012. Both observational studies and prospective therapeutic trials were eligible for inclusion.

### Information sources

We identified published studies using PubMed, Google Scholar, Web of Science, African Journals Online, and private archives. Where published bibliographies of the recovered studies were found to contain promising citations (including grey literature) not included in online searches, these papers were obtained, whenever possible, and screened for inclusion in the meta-analysis. Contact with authors who had not presented full test performance data in their papers yielded individual level data sufficient to include two additional survey studies.

### Search strategies

We started with the wildcard keywords schistosom# and bilharz# (*e.g.*, ‘*Schistosoma*’, ‘schistosomiasis’, ‘schistosome’, ‘bilharzia’, or ‘bilharziasis’), combined with ‘dipstick’, ‘hematuria’, ‘chemical reagent strip(s)’, ‘reagent strips/diagnostic use’, ‘bandelettes/urinaires’ and the terms ‘comparative study’, ‘evaluation study’, ‘diagnosis’ or ‘mass screening’. As relevant articles were identified, we broadened our search by accessing additional titles through the online databases' automated ‘related articles’ links. Full titles and abstracts were recovered for initial review for inclusion.

### Study selection

Two reviewers independently screened each study recovered in our search lists for inclusion in the systematic review. Those studies found suitable for inclusion were then obtained from online or library sources for full-text review. Where a single report contained data on multiple individual community surveys, each survey was also separately abstracted for inclusion in some of the sub-group comparison analysis. Both observational and prospective studies were considered eligible for inclusion in the overall analysis. We excluded studies where comparator parasitological diagnosis was not reported, or when the data on individual level hematuria and egg count status was not sufficiently detailed to confirm the reported sensitivity and specificity of dipstick testing. Cases of duplicate publication or extended analysis of previously published studies were also excluded.

### Data collection process

Included papers were abstracted and their relevant features entered into a purpose-built database created in Microsoft Access 2010 software (Redmond, WA). In addition to full citation information and year of publication, information was collected on the country and region where the study was performed, the target population studied (children, school-age children, adults, community, etc.), their prior treatment status, the sex and age distribution of included subjects, study size, local infection prevalence in general, dipstick manufacturer, definition of hematuria (dipstick cutoff value, i.e., ≥trace, 1+, 2+, or 3+, as scored by study technicians, based on the manufacturer's instructions using a color chart supplied with the dipsticks), method of parasite egg detection, number of urine samples tested, and the study's definition of ‘light infection’. Whenever possible, raw data for true positives, false positives, true negatives, and false negatives were extracted from the study's text or tables, or back calculated from the included summary data. These values were entered into separate 2 by 2 tables and the dipstick diagnostic performance was reconfirmed for each reported survey. Data entries were fully verified by a second reviewer before final data analysis was carried out. As practiced in a previous systematic quantitative review of *S. haematobium* diagnostics [22], we allowed each included research report to contribute multiple observations to the analysis, *i.e.*, data reported from individual communities or schools were included as independent observations.

### Summary measures

Once study eligibility was determined, test results from each study and sub-study were entered into RevMan 5 software (available from the Cochrane collection at http://ims.cochrane.org/revman) for calculation of study prevalence, sensitivity, specificity, positive predictive value and negative predictive values, along with their confidence intervals. Dipstick performance was assessed initially according to study size, infection prevalence, and region, by visual comparison of forest plots. Summary Receiver Operating Curves (SROC), were also graphed using the RevMan5 analysis module.

### Synthesis of results

Heterogeneity among studies was determined using Higgins's and Thompson's I^2^ statistic. In exploratory data analysis, multivariable meta-regression examined the impact of additional factors (study era, dipstick brand, age grouping (i.e., school age vs. community), male∶female ratio, and world region) on diagnostic odds ratios associated with dipstick testing. For this analysis, a Moses-Shapiro-Littenberg method study size-weighted model of test performance in the ROC plane was implemented in Meta-DiSc Software v.1.4 (provided by Hospital Ramon y Cajal, Madrid at its website, http://www.hrc.es/investigacion/metadisc_en.htm) [25].

Final pooled summary estimates for sensitivity and specificity were calculated by using hierarchical summary ROC (HSROC) regression following a Bayesian Monte Carlo Markov Chain approach as described by Dendukuri, et al, [26] and implemented using their programs (available at http://www.nandinidendukuri.com) in SAS v.9.3 statistical software. This approach was selected because of its ability to adjust for heterogeneity by assessing within- and between-study variability in test performance, while also including the effects of imperfect sensitivity and specificity of the various different reference tests (*e.g.*, egg counts by filtration, centrifugation, or sedimentation) included in the meta-analysis. Sub-group analysis examined the impact of i) population prevalence of *S. haematobium* egg positivity in urine, ii) class of intensity of infection (‘light’ vs. ‘heavy’, as determined in each study using measured egg counts/10 mL urine (see Results section, below, for details), and iii) pre- vs. post-treatment status, on test accuracy in terms of sensitivity and specificity.

## Results

### Study selection

**Figure 1:**
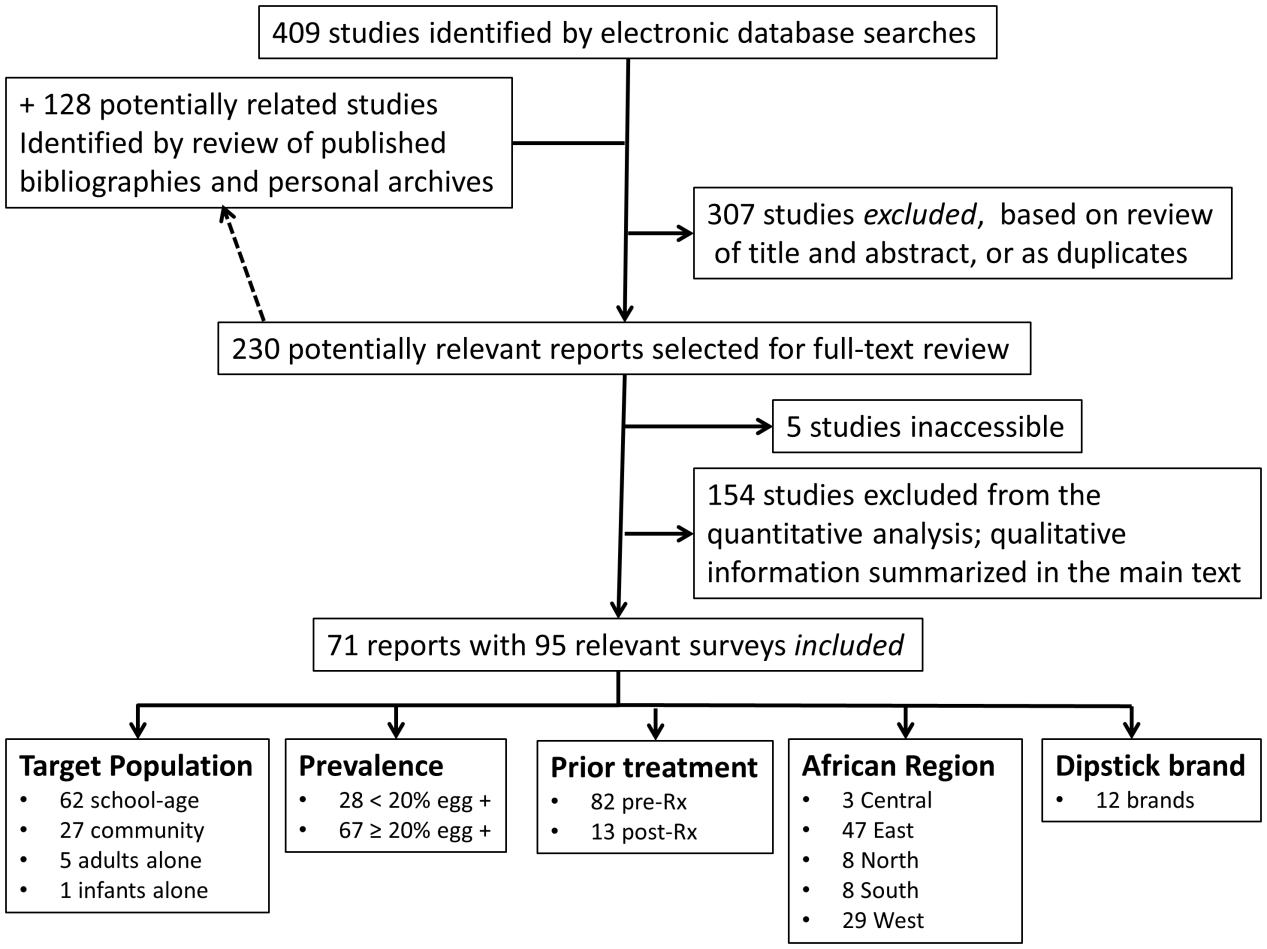
Flow chart of study search and selection strategy.

Our search strategy recovered a total of 537 listings, of which 409 were identified by online database searches, and an additional 128 were found through searches of bibliography listings and private archives (see Figure 1). Of these, 307 were excluded after initial review as not relevant to the area of dipstick diagnostic performance, or as duplicate entries. 230 were selected for full review, and 71 reports, containing data on 95 separate surveys, were included in the meta-analysis (see supporting information files, Table S1-Included Studies and Table S2-Excluded Studies, for study listings and their citations). Details of inclusion and exclusion criteria for the analysis are provided in the Methods section, above.

### Study characteristics

All studies were identified through peer-reviewed publications. Eighty-eight were in English, and seven were in French. Because criteria for performance and reporting of clinical trials have continued to evolve over the last two decades, we included study age as a covariate that might potentially influence results. Sixty-four (67%) of the 95 included studies were reported after 1989, whereas 31/95 were from the pre-1990s era (1979–1989). Five studies focused on adults alone, one focused on infants alone, 27 were reported as community-based surveys, and 62 were performed with school-aged children alone (Figure 1). Included studies ranged from 5%–100% female participation (where reported, the median female participation was 50%, IQR = 47–56%), and only 3/95 (3%) of studies excluded women over 11 years of age. Geographic variation in parasite strains has also been suggested as a source of variation in infection-associated morbidity—among the included studies, 47 were from East Africa, 29 from West Africa, 8 from southern Africa, 8 from North Africa, but only 3 from central African countries.

Eighty-two of the reported studies were performed before any mass anti-schistosomal treatment had been given to the study population, whereas 13 of the included studies were performed on previously treated populations, usually at a 1 year post-treatment interval follow-up. There were 8 paired surveys performed on the same populations before and after treatment.

**Figure 2:**
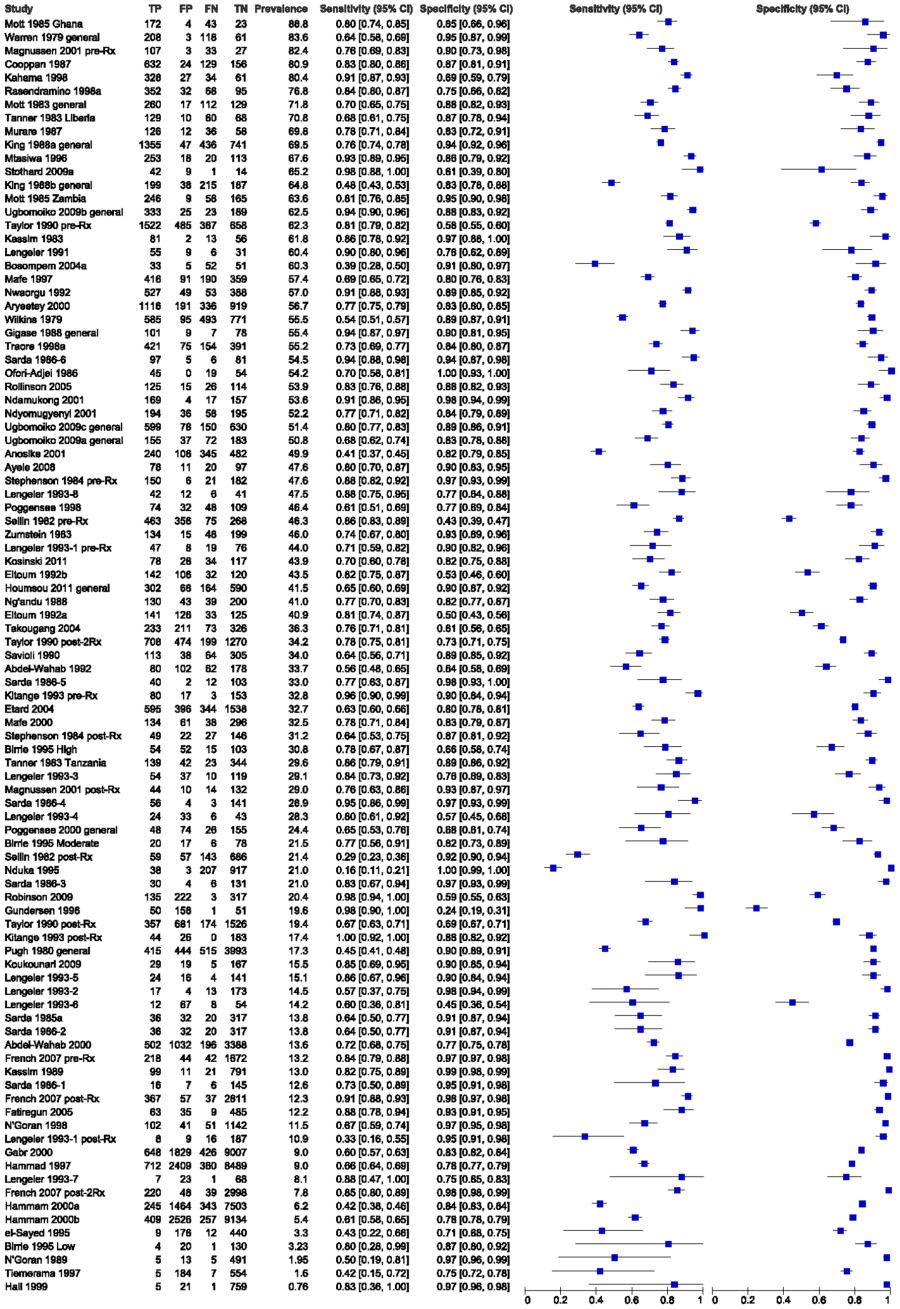
Forest plot of dipstick sensitivity and specificity according to prevalence of egg-positive urines in study. All included studies are listed in descending order of study prevalence of egg-positive urines, starting with their brief citation, then individual data on observed true-positives, false-positives, false-negatives, true-negatives, then study population prevalence. Center columns indicate calculated sensitivity and specificity for each study, with their respective 95% confidence intervals. The forest plots along the right-hand side graphically indicate observed dipstick sensitivity and specificity for each study (blue squares), while the horizontal black lines indicate the 95% confidence interval for each value.

Egg output in subject urine was detected by filtration in 83/95 studies, by centrifugation in 8 studies, and by sedimentation in 3 studies. One study did not report on their egg detection technique. Twenty-seven studies reported on dipstick performance among subjects with ‘light’ intensity infection. Light intensity infection was most commonly defined as <50 or 51 eggs per 10 mL aliquot of urine (N = 29), but varied in definition from <11 eggs to <500 eggs per 10 mL. [N.B. in published studies, the general definition of ‘light infection’ shifted from ≤100 eggs/10 mL urine in the 1970s, to ≤50 eggs/10 mL in the 1980s. For this paper, any studies using a definition of ‘light’ as ≤100 eggs/10 mL (or lower) were included for the light infection subgroup analysis] Prevalence of urine egg positivity among the tested study populations ranged from 0.76% to 88% (median 34%, IQR = 15–55%, see Figure 2). Studies in 29/95 of included reports had prevalence of egg positivity (<20%) and these were analyzed as a separate ‘low prevalence’ subgroup in some of the analysis below. Urine egg testing was based on a single daily urine in 79/95 of the included studies, multiple (2–6) daily urines were tested in 10/95 studies, whereas 6 studies did not provide specific information about the number of urines tested. Of the studies with multi-day testing, most provided results for the first day's egg count and heme dipstick testing. To harmonize their inclusion in the meta-analysis, only these first day results were included in the analytic database used in this paper's results. For performance analysis of multi-day testing, readers are referred to Savioli at al. [27], and Tiemersma et al., [28]

### Risk of bias

The surveys selected for inclusion in the meta-analysis and meta-regression were felt to be representative, *i.e.*, those used in screening of schools, whole communities, school-age children, or adult women, within target communities. Partial or differential verification bias did not apply to these studies. However, in nearly all studies, data were **not** available on non-participation, on delays in testing, or on the number of uninterpretable results, nor were there descriptions of efforts to mask the results of the two tests being compared during the performance of the studies.

### Results of studies

#### a. Detection of egg positive urine

Across all 95 included studies, there was a broad range of dipstick performance for the diagnosis of *S. haematobium* infection; for the detection of egg positive urine, reported sensitivity ranged from 16 to 100% and reported specificity ranged from 24–100% (Table 1). As expected, heterogeneity values across the range of studies were quite high (*I^2^* values>95%) for the total studies and various sub-groups tested. Forest plots for studies ranked by their subject population's prevalence of egg-positive subjects (Figure 2) or by study size (supporting information, Figure S1) did not indicate any clear trends in dipstick performance according to those two factors.

**Table 1:** Diagnostic performance of dipstick detection of microhematuria as a proxy for egg detection in the urine.

Group/Subgroup	Number of studies	Dipstick Diagnostic Performance	Range of Reported Values	Summary Estimate by HSROC^a^	95% CI^b^
**All studies**	95	Sensitivity^c^	16–100%	81%	(79, 83%)
		Specificity	24–100%	89%	(87, 92%)
**High prevalence locations**	66	Sensitivity	16–98%	80%	(78, 83%)
		Specificity	43–100%	86%	(82, 90%)
**Low prevalence locations** d	28	Sensitivity	42–100%	79%	(75, 84%)
		Specificity	24–99%	90%	(84, 94%)
**Light intensity subgroup**	25	Sensitivity	16–87%	**65%**	**(58, 72%)**
		Specificity	50–97%	82%	(76, 90%)
**Prior to treatment**	81	Sensitivity	16–98%	81%	(77, 84%)
		Specificity	64–98%	90%	(85, 93%)
**Post treatment**	13	Sensitivity	29–100%	**72%**	**(61, 78%)**
		Specificity	24–100%	87%	(81, 94%)

a summary estimate and.

b 95% credible interval derived by Bayesian hierarchical summary receiver operating curve (HSROC) analysis [26].

c Sensitivity and specificity for dipstick detection of egg-positive urine.

d Prevalence below 20%.

**Figure 3:**
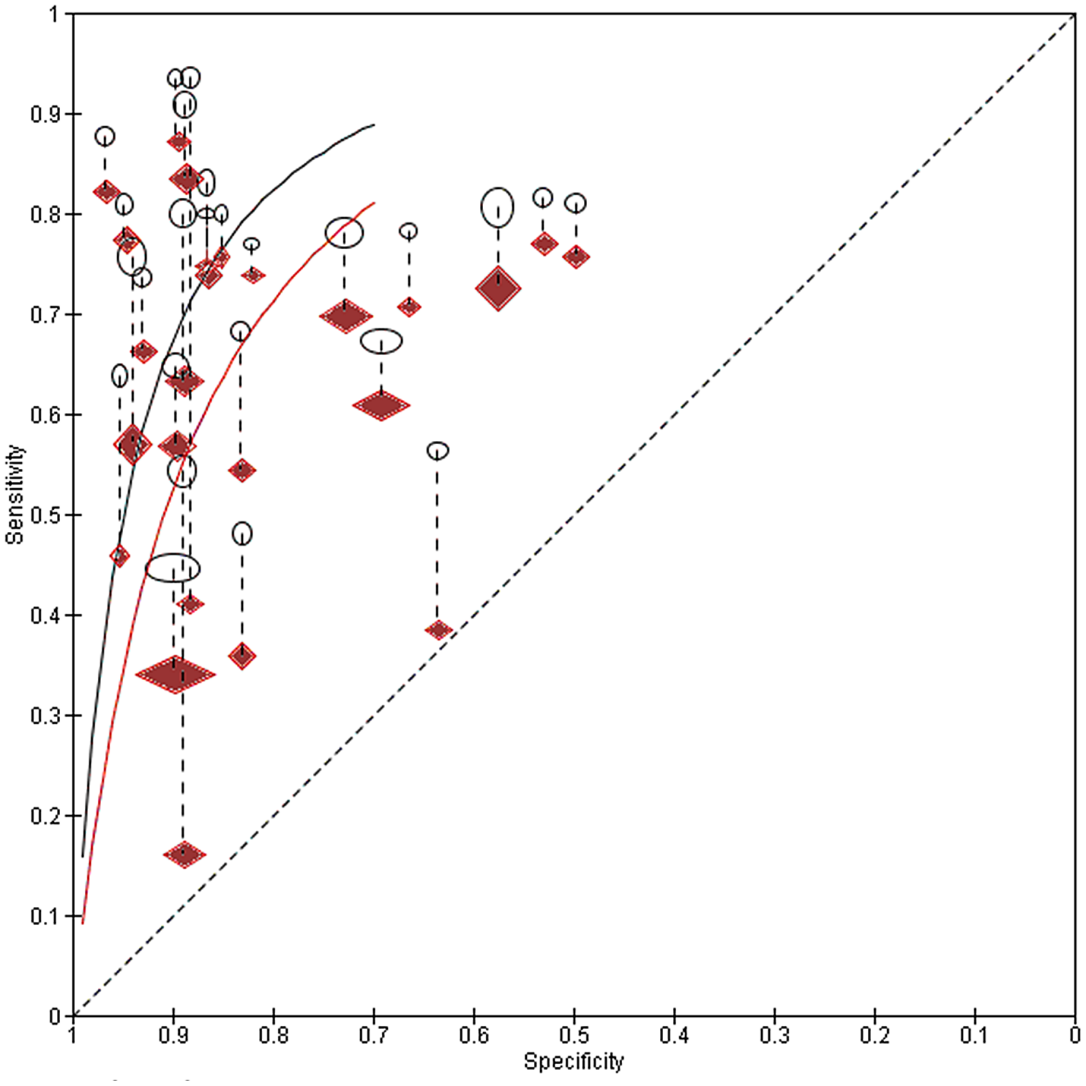
Receiver-operating characteristics (ROC) for dipstick performance in detection of *S. haematobium* egg-positive urine. Sensitivity/specificity of dipstick performance among sub-populations having light intensity infection (solid red diamonds), as compared to dipstick test performance reported for the total population in that study (open black ellipses). The symbol for each study is proportional to study size. Lines with long dashes link the data points for each study pair (N = 25 pairs). The short-dashed diagonal line indicates the line of test non-discrimination (i.e., worst performance). The two smooth curves indicate the weighted summary ROC curves for each group—total population in black, and light-intensity subgroup in red.

**Figure 4:**
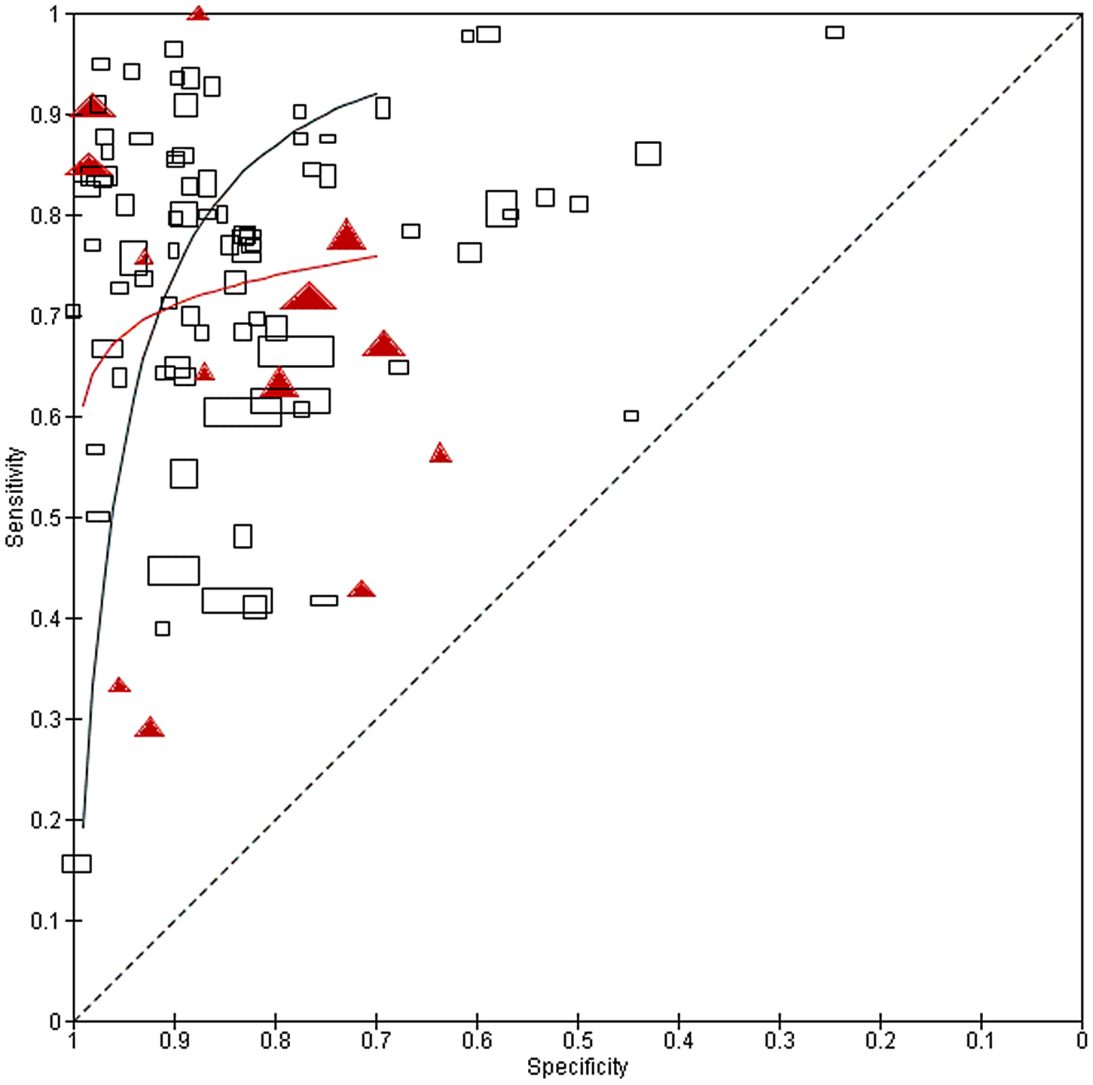
Performance of dipsticks for detection of *S. haematobium* egg-positive urine in pre- and post-treatment populations. ROC plot of sensitivity/specificity of dipstick performance among untreated populations, as compared to populations having had previous anti-schistosomal treatment. Untreated populations are indicated by open black rectangles, while previously treated populations are indicated by solid red triangles. Symbols for each study are proportional to study size. Curved lines indicate the summary performance curves estimated by HSROC for each group—for untreated populations in black (N = 81) and for treated populations in red (N = 13).

**Figure 5:**
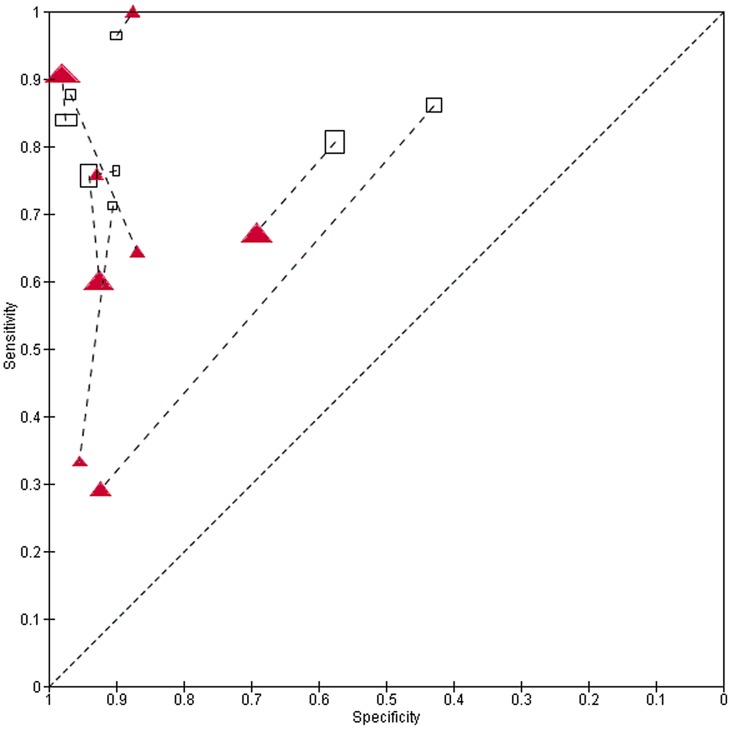
Paired shifts in dipstick performance from before treatment to after treatment in the same population. Untreated populations are indicated by open black rectangles, while treated groups are indicated by solid red triangles. The symbol for each study is proportional to study size. Lines with long dashes link the data points for each study pair (N = 8 pairs).

Subsequent meta-analysis of dipstick detection of egg positive urines indicated no reduction in sensitivity among low prevalence (<20% egg-positive) populations (Table 1). However, where studies reported on dipstick performance for their subgroup of subjects having ‘light intensity’ infections, or among populations who had previously received treatment, dipstick sensitivity was significantly lower (65% and 72% sensitivity, respectively, for light intensity and post-treatment status, as compared to an estimated 81% (95% CI: 79, 83%) sensitivity for study populations in general, Table 1). Specificity, however, was not significantly different for these two subgroups. Figure 3 graphs the relative performance of dipsticks for total vs. light intensity populations for the 25 studies with paired data, and indicates the estimated HSROC curves for these two groups. Figure 4 shows the relative performance of dipsticks for detection of egg-positive urine, contrasting pretreatment surveys and post-treatment populations. Figure 5 shows the shifts in performance in 8 studies having paired surveys in the same populations that were examined pre- and post-treatment. The net change in performance was very mixed in these paired studies—four studies indicated decreased sensitivity but increased specificity after treatment, two showed both decreased sensitivity and specificity, while two showed *increased* sensitivity and mostly unchanged specificity after treatment.

**Figure 6:**
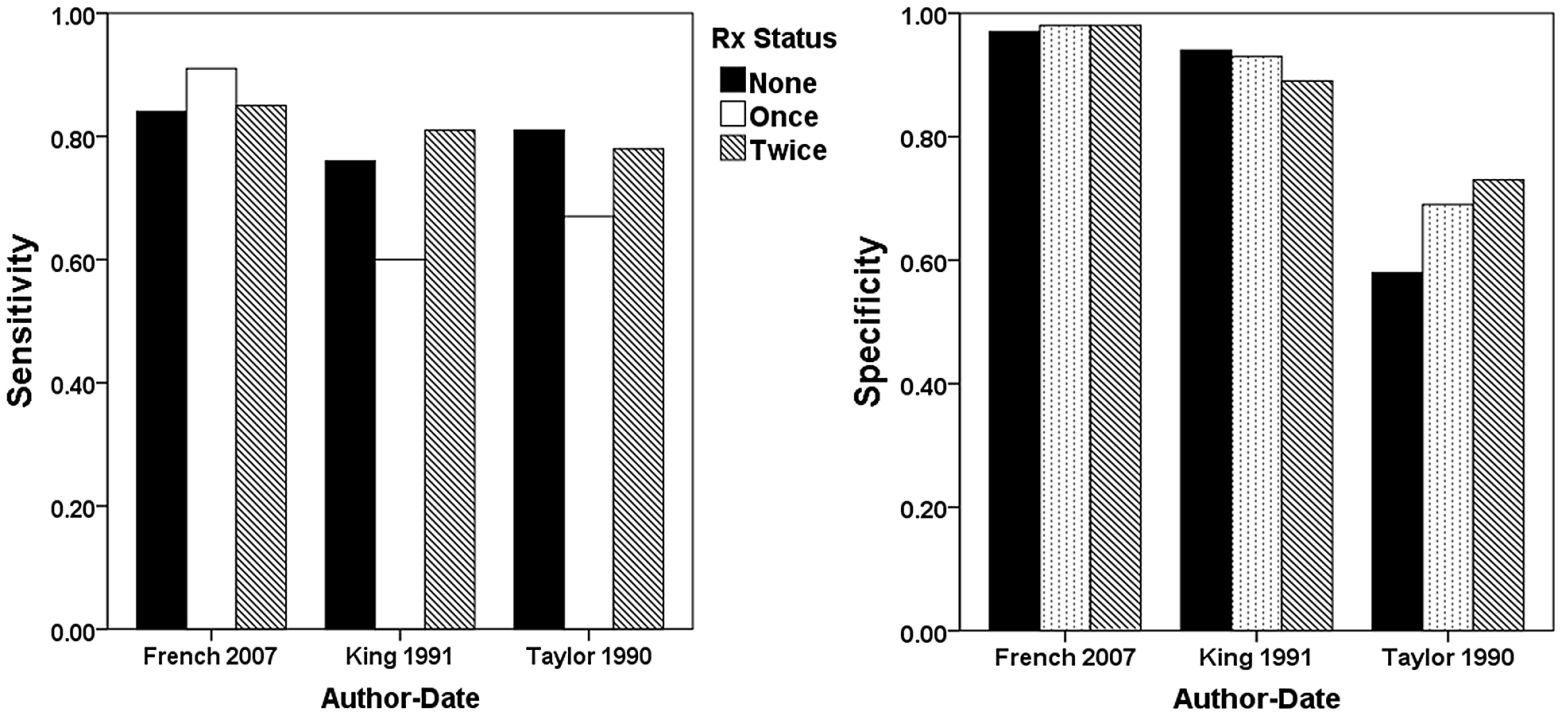
Dipstick performance before treatment and after one or two rounds of therapy. Shown are the dipstick diagnostic characteristics in the three studies that provided data on performance after multiple rounds of school age chemotherapy. Sensitivity, as described for the individual studies [9], [29], [30], is shown in the left panel and specificity in the right panel, with treatment status indicated as follows: solid black bars, untreated children; open bars, children previously treated one time; hatched bars, children previously treated twice.

Three studies [9], [29], [30] provided data on dipstick performance among school age children after two rounds of treatment. Figure 6 shows their reported dipstick sensitivity and specificity for detecting egg-positive urines after one and two rounds of treatment, as compared to pre-treatment values. After the first round of treatment, dipstick sensitivity declined in two out of three of the projects. It was noted, however, after two rounds of treatment, dipstick sensitivity returned to baseline (pretreatment) performance in all studies (Figure 6). In these studies, dipstick specificity remained near baseline or increased [9] following treatment.

#### b. Dipstick estimation of the presence of infection

There are recognized limitations to using egg detection for diagnosis of active *S. haematobium* infection, particularly if infection intensities are light, or if a program relies only on one single daily urine examination in its testing strategy [9], [23], [27], [31]. To address this issue, our hierarchical Bayesian Monte Carlo Markov Chain summary estimation of dipstick ROCs (HSROCs) was redone assuming that urine egg-detection is not a true gold standard (*i.e.*, that realistically, it has less than perfect sensitivity and specificity) [26]. In this re- analysis, the premise was that both dipstick heme positivity *and* microscopic egg positivity were separately indicative of active *S. haematobium* infection, that neither was a perfect indicator, and that the relative diagnostic performance could be well-estimated by HSROC parameter fitting for both tests. The latent construct of ‘probable infection’ was thus defined by a mixed input of data from both tests.

Table 2 summarizes the estimated sensitivity and specificity of urine heme dipsticks for the combination of overt (egg-positive) or occult (egg-negative) *S. haematobium* infections that were active in the surveyed populations. Of special note, we found that where local prevalence was lower (<20%), HSROC estimated that dipstick sensitivity was decreased (but specificity was increased) when compared to dipstick performance in high prevalence areas. In contrast to the analysis done in Table 1, (in which egg counts had been taken as a gold standard of infection), in the reanalysis, dipstick performance for light intensity and for post-treatment subgroups was not significantly different from performance seen when all intensities were combined, or when only pre-treatment subgroups were tested (Table 2).

**Table 2:** Estimated performance of microhematuria as proxy for active *Schistosoma haematobium* infection.

Group/Subgroup	Dipstick Diagnostic Performance	Summary Estimate by HSROC^a^	95% CI^b^
**All studies**	Sensitivity^c^	82%	(80, 84%)
	Specificity	97%	(95, 98%)
**High prevalence locations**	Sensitivity	92%	(89, 94%)
	Specificity	87%	(83, 89%)
**Low prevalence Locations** d	Sensitivity	79%	(73, 83%)
	Specificity	98%	(95, 99%)
**Light intensity subgroup**	Sensitivity	82%	(74, 92%)
	Specificity	96%	(91, 98%)
**Prior to treatment**	Sensitivity	83%	(80, 85%)
	Specificity	96%	(94, 98%)
**Post treatment**	Sensitivity	79%	(68, 92%)
	Specificity	95%	(84, 99%)

a summary estimate and.

b 95% credible interval derived by Bayesian hierarchical summary receiver operating curve (HSROC) analysis [26].

c Sensitivity and specificity for detection of active *S. haematobium* infection, as identified by a combination of egg count and hematuria status information, assuming both tests are imperfect [26].

d Prevalence below 20%.

For comparison, when this same ‘latent construct of infection’ approach was used to assess the performance of egg microscopy as a diagnostic test for *S. haematobium* infection, sensitivity for detection was 81% (95% CI: 78, 85%) across all studies (N = 96). It was significantly lower, at 51% (95% CI: 40, 62%) in low prevalence areas (N = 28), and at 58% (95% CI: 38, 76%) in previously-treated groups (N = 13). It was estimated at 88% (95% CI: 81, 95%) for subgroups with light infections (N = 25). In each case, the estimated test specificity for egg counting was >98%.

#### c. Meta-regression

Many factors are postulated to affect the performance of dipstick testing and urine egg counting as diagnostics for *S. haematobium* infection. Beyond local prevalence and intensity of infection, these covariates were postulated to include: choice of dipstick cutoff (≥trace or ≥1+), dipstick brand, era of study, subject age, female participation rates, regional variations in infection-associated morbidity, and mode of egg detection (see supplementary Figures S2, S3, S4). Of note, 24/95 of studies did not indicate the cutoff that was used for scoring a dipstick positive for hematuria. In multivariable meta-regression, we found that the survey's targeted age group (school age vs. community) had the strongest influence on dipstick diagnostic performance, even after adjustment for other factors. The relative diagnostic Odds Ratio (rDOR) was 3.16 (95% CI: 1.89, 5.29, *P*<0.001) for school age children surveys compared to community surveys (Table 3), suggesting a much better test performance among children as a group, independent of prevalence, treatment status, and other factors included in the model. In this analysis, there was a suggestion of significant differences among dipstick brands in terms of diagnostic performance. However, this phenomenon proved not to be consistent in subsequent subgroup analyses when stratifying by age or mode of parasitological diagnosis (supplementary tables S3, S4). A factor that was consistently related to dipstick performance in all groups was location—studies in North Africa had consistently and significantly lower diagnostic performance for dipsticks than elsewhere in Africa (rDOR = 0.22 (95% CI 0.09, 0.53, *P*<0.001)), independent of other factors (Table 3 and supplementary Tables S3 and S4).

**Table 3:** Exploration for sources of heterogeneity.

Covariate	Singly-adjusted			Multiply-adjusted^a^		
	RDOR	95% CI	P value	RDOR	95% CI	P value
Low Prevalence	1.15	0.6, 2.19	0.67			
Post-Rx	0.65	0.28, 1.49	0.30			
Brand 1	**0.27**	**0.08, 0.90**	**0.033**			
Brand 2	0.82	0.31, 2.18	0.69			
Brand 3	–b	–	–			
Brand 4	–	–	–			
Brand 5	0.70	0.34, 1.42	0.32	**0.50**	**0.27, 0.92**	**0.026**
Brand 6	0.35	0.05, 2.28	0.27	0.27	0.06, 1.25	0.094
Brand 7	**2.39**	**1.16, 4.91**	**0.019**			
Brand 8	0.39	0.12, 1.19	0.098	**0.26**	**0.10, 0.67**	**0.006**
Brand 9	1.93	0.89, 4.19	0.094			
Brand 10	1.33	0.27, 6.63	0.724			
Brand 11	0.37	0.05, 2.45	0.30			
Brand 12	1.87	0.55, 6.34	0.31			
Egg detection	0.47	0.22, 1.03	0.06			
Central Africa	2.69	0.54, 13.5	0.225			
East Africa	**2.03**	**1.16, 3.54**	**0.013**			
North Africa	**0.17**	**0.07, 0.46**	**<0.001**	**0.22**	**0.09, 0.53**	**<0.001**
South Africa	0.58	0.23, 1.48	0.249	**0.33**	**0.15, 0.73**	**0.007**
West Africa	0.89	0.47, 1.70	0.731			
School-age only?	**3.38**	**1.95, 5.84**	**<0.001**	**3.16**	**1.89, 5.29**	**<0.001**
Study Era	0.88	0.61, 1.26	0.474			
Dipstick Threshold	1.08	0.76, 1.53	0.68			

Relative covariate effects (singly- or multiply-adjusted) on diagnostic Odds Ratios for dipstick diagnosis of egg-positive urine among all included studies (N = 94).

a A best fit reduced multivariable adjusted model was selected by stepwise backward removal of non-significant cofactors from a full model containing all covariates. The final working model presented here was selected when all variables in the model were either statistically significant or biologically plausible and marginally significant.

b The dashes indicate insufficient data in this category for an estimate.

#### d. Risk of bias across studies

None of the included studies reported in detail on their own inclusion/exclusion criteria, subject adherence, or masking of observers during comparator diagnostic testing. Therefore, the influence of these factors our overall estimations could not be examined. We did not observe any trends in outcomes relative to study size or year of publication. All included survey datasets were derived from published papers, except for two sets of supplemental data that were provided by authors contacted for more detail on their follow-up studies. Ultimately, but not by design, the studies included in the meta-analysis were only published in English or French and all came from 21/44 of the *S. haematobium-*endemic countries in Africa (21/53 of endemic countries worldwide [1]). Tanzania (34 studies), Nigeria (15 studies), Egypt (7), Kenya (6) and Ghana (6) provided the large majority of the 95 included studies, while other countries were less well represented in the data analyzed.

## Discussion

Our meta-analysis indicates that commercial dipsticks, designed for rapid detection of heme in the urine, can provide an effective proxy for detection of *S. haematobium* infection in disease-endemic areas. The study populations included in our meta-analysis were felt to be representative of *S. haematobium* endemic communities likely to be targeted in regional and national control programs. Overall, dipstick performance showed 81% sensitivity and 89% specificity for detection of egg positive urines, and an estimated 82% sensitivity and 97% specificity for detection of active *S. haematobium* infection, as estimated via the combination of dipstick and egg count results. Whereas dipsticks were less sensitive in detecting egg-positive urines among post-treatment studies and among subject sub-groups with lower intensity infections (Table 1, Figures 3 and 4), evaluation of paired pre- and post-treatment studies did not show a consistent post-treatment effect on dipstick diagnostic performance (Figure 5). When the limitations of egg-count diagnosis of *S. haematobium* infection were taken into account, diagnostic performance was not significantly different between pretreatment and post-treatment study populations (Table 2).

Significant differences in dipstick diagnostic performance were noted based on the age range of included survey subjects (school-age vs. community-wide). In addition, studies from North Africa were found to have significantly lower dipstick performance, independent of age, local prevalence, treatment status, and other measured co-factors, suggesting a possible differential in risk for *Schistosoma-*associated morbidity between North African and sub-Saharan populations [32], [33]. Previous reports have commented on differences in dipstick performance between Egypt/Ghana *vs.* Zambia [34]and between Liberia and Tanzania [35] in parallel survey studies.

There have been continuing concerns about ‘false positives’ for heme dipsticks, resulting in an apparently low specificity in some populations [36]–[40]. In their 2004 mathematical synthesis of dipstick performance, van der Werf and de Vlas [22] estimated an average 18% ‘false positive’ rate for microhematuria for the diagnosis of *S. haematobium* (i.e., heme positive but egg negative), based on an assumed low likelihood of infection in this class of patients. They presumed that this 18% had to be due to non-schistosome causes, because “…at low prevalence of infection…only few or no cases have an infection intensity high enough to cause morbidity.” [22] This assumption, *i.e.*, that low-intensity infection is non-morbid, has been critically challenged over the last decade [41]; with the use of complementary, more sensitive diagnostics, it now appears that many heme-positive egg-negative subjects in endemic areas are, in fact, *S. haematobium-*infected [27], [42]–[45]. In a ‘low prevalence, low-intensity’ community in Nigeria (9.5% egg positive, but 52% heme positive) infection prevalence determined by *Schistosoma-*specific PCR was, in fact, 93–98% [46]. As an unmeasured cofactor, differential circadian variation in egg output and hematuria [47], [48] might account for some of the variation among studies observed in our analysis. In non-endemic areas such as North America, a cumulative 3% of ambulatory school children have hematuria between 6 and 12 years of age [49] and 3–13% among otherwise healthy adults [50], [51], suggesting that in *S. haematobium*-endemic areas, the balance of 5–15% of the population with heme-positive/egg-negative urines actually do suffer from active schistosomiasis that is not detected by egg counting. In summary, the specificity of dipstick heme diagnosis is likely to be more specific in *S. haematobium* transmission zones than previously assumed [22].

Contamination of urine by menses, or by inflammation from bacterial cystitis, sexually-transmitted infection, or genital mutilation, has been suggested as a source of false positive dipstick diagnosis among girls and women [36], [37], [52]. Studies of adult women, particularly focused on menstrual status, indicate that there is a risk of false positive dipstick results based on this factor. In *S. haematobium*-endemic communities, prevalence of heme-positive urine among women who are urine egg negative is 33–53% during menses, and 10–17% when not having menses [52]. However, the current meta-analysis did not find evidence that the proportion of participating females in a survey had an effect on overall dipstick performance at the population level. It is possible that women having menses self-exclude from screening surveys for cultural, religious, or personal reasons [36]. In practice, the effect of menses has been minimal in surveys of 4–20 year olds South Africa [53], in community-wide surveys in Zanzibar [27], [54], [55], and among adult age groups (>15 years old) screened in communities in Cote d'Ivoire [56].

Where studied, subject age has been suggested to have an important effect on dipstick diagnostic performance. In several studies, for each infection category, children consistently had a greater prevalence of hematuria than adults with the same infection intensity [57]–[61]. The hypothesis is that adults experience less inflammation or release less blood in response to parasite egg deposition around the urinary tract. Koukounari et al. [62] specifically observed that dipstick sensitivity for detection of *S. haematobium* infection was not as good among adults >50 years old in village surveys in Ghana. By contrast, Eltoum, et al. [63] in Sudan, and Poggensee, et al. [52], in Tanzania found disproportionately higher rates of hematuria among older adult groups despite their lower prevalence of egg-positive urines.

Although there was a high degree of heterogeneity in dipstick test performance across all studies, this sort of variation is common in comparisons among diagnostic trials [64]. The HSROC approach used to estimate the summary sensitivity and specificity values reported in Tables 1 and 2 allowed for the influence of both inter- and intra-study variability, and is believed to provide a robust estimation of overall dipstick performance [26], [64]. Bayesian HSROC also allowed us to examine the likely sensitivity and specificity values for dipsticks given the imperfections of urine egg detection methods used as the comparison diagnostic test [26]. Considering the lack of a true gold standard for diagnosis of *S. haematobium* infection [27], the analysis in Table 2 allows us to infer that dipsticks retain their validity as a diagnostic tool, even when eggs in the urine are scarce or become so after a round of therapy. In this latent construct analysis, for low prevalence areas the estimated sensitivity for detection of active *S. haematobium* infection was higher for dipsticks (79%) than for egg detection (51%) while specificity was the virtually the same (98–99%). In post-treatment populations, sensitivity for dipsticks was 79%, compared with 58% for egg-detection. This suggests that reagent dipsticks may be the preferred community-level diagnostic screening tool as programs reduce parasite prevalence and move towards elimination.

This meta-analysis had several limitations. Risk of bias in the included studies was for the most part, unknown. Many of the included studies did not aim to evaluate dipstick performance as their primary outcome, but rather reported on detected hematuria as a marker of *S. haematobium-*associated morbidity. No study reported in much detail on inclusion/exclusion criteria, subject adherence, or on masking of observers to the results of comparator diagnostic tests. As such, these studies did not meet the usual criteria for ‘high-quality’ diagnostic test clinical research [64]. However, we recognize the limitations of such studies in this underfunded area of neglected tropical diseases research–given the need for a working summary of the ‘state of the art’ for *S. haematobium* screening, we elected to include all available data from comparable field studies meeting our inclusion criteria. Across all studies, we recognize the possibility that certain countries were under-represented, as evidenced by lack of data inputs from lusophone Africa and an absence of data from the Arabian Peninsula or other Middle Eastern countries, a circumstance that could have biased our assessment.

While there was broad variation in the performance of heme dipstick diagnosis of *S. haematobium* infection, our meta-analysis could identify certain important themes:

- ***As a predictor of egg detection in a urine specimen***, dipsticks are somewhat less sensitive for low-intensity infections, or when populations are surveyed after treatment (Table 1). The latter effect is presumably due to the fact that high-intensity infections become much less common in treated communities.
- ***As a predictor of probable*** **S. haematobium** ***infection*** (reflecting on known insensitivity of microscopic egg counting methods for diagnosis of low-intensity infections) we find that the dipstick retains >79% sensitivity for detection of infection even in low prevalence and post-treatment areas. Believing that the majority of hematuria in low-intensity infection and post-treatment communities is still caused by *S. haematobium* infection, dipstick diagnosis remains quite accurate, although less sensitive and more specific than when used in high prevalence areas (Table 2).
- ***The performance of the dipstick is significantly better in surveys of school-age children than among adults or in surveys of communities at large*** (*i.e.*, those that include both children+adults). In both school and community surveys, dipstick performance was consistently less strong in North African locations.
- ***There was a good deal of variation in dipstick performance from site to site*** and from study to study, and our stratification by age group, location, or urine egg count methods did not resolve this observed heterogeneity among studies. It is important for quality assurance, therefore, to periodically validate local dipstick performance as part of monitoring and evaluation of control program implementation [9], [53], [65], in particular, assuring that the dipsticks are properly stored, handled, and read [57], [66].

Overall, on an *individual patient basis*, it appears from our meta-analysis that, after treatment has been given, dipsticks become less efficient in detecting a person who has eggs in his or her urine. However, given the insensitivity of egg counting for low intensity infections, dipsticks may ultimately prove more sensitive for detection of low-level persistent *S. haematobium* infection than testing for egg output on any given single day. In the studies included in our review, dipstick performance after treatment was seen to change in several different ways, depending on location. This finding points to the need for formal detailed diagnostic trials against a truly sensitive and specific ‘gold standard’ of active *S. haematobium* infection, particularly after one or more rounds of treatment have been given. This applies particularly to pre-school children and adults, for whom egg output may be relatively limited. Ongoing studies combining anti-parasite serologies and high-sensitivity detection of circulating parasite antigens should help to clarify the actual diagnostic performance of dipsticks and egg-filtration in detecting early and low-intensity *S. haematobium* infections. If and when population-based treatment is restricted to only ‘infected’ persons [62], neither the urine egg count nor the dipstick heme test could be considered sufficiently sensitive to accurately identify a full fraction of ‘true infections’ to effect full prevention of morbidity [24].

On a *population basis*, dipsticks remain very valid tools for use in public health campaigns for control of urogenital schistosomiasis. As current preventive chemotherapy campaigns move forward, the prevalence and infection intensity profiles of *S. haematobium*-endemic communities undoubtedly will change. Overall, based on our synthesis of the available data, we expect that the urine dipstick for detection of microscopic hematuria will retain its usefulness as a tool for estimation of *S. haematobium* prevalence in treated and untreated communities [21], [29], [67]–[69]. The strong link between infection, urinary tract ulceration, and resultant hematuria means that the inexpensive and rapid detection of urine hematuria can continue to be a useful estimator of local prevalence. As such, dipsticks will serve as useful adjuncts for monitoring the impact of schistosomiasis control programs on prevalence, and can guide us in determining the need for further program interventions.

## Supporting Information

Checklist S1 **PRISMA checklist.**
(DOC)

Figure S1 **Forest plot of dipstick sensitivity and specificity according to size of the study population.**
(TIFF)

Figure S2 **Diagnostic performance according to cutoff standards for the semi-quantitative heme reaction on a dipstick pad.** Exploratory Moses-Shapiro-Littenberg SROC analysis curves indicate the relative performance of dipsticks for diagnosis of egg-positive urine, comparing studies using > = trace (red diamonds) *vs.* > = 1+ readout (green squares) *vs.* > = 2+ readout (blue triangles) for their cutoff criterion to define ‘hematuria positive’. Those studies that did not indicate a cutoff are shown by the black ellipses. Corresponding ROC curves are shown in the same colors. Symbol size is proportional to study enrollment.
(TIF)

Figure S3 **Diagnostic performance according to the age groups included in a dipstick diagnostic study.** Exploratory Moses-Shapiro-Littenberg SROC analysis curves indicating the relative performance of dipsticks for diagnosis of egg-positive urine, comparing studies performed on communities at large (black ellipses, black line), school age children only (red diamonds, top red line), adults only (blue triangles, blue line), or clinic-based adults (green-squares, middle green line). Symbol size is proportional to study enrollment.
(TIF)

Figure S4 **Dipstick diagnostic performance for detection of egg-positive urine according to the method of egg detection.** Exploratory Moses-Shapiro-Littenberg SROC analysis curves indicating the relative performance of dipsticks for diagnosis of egg-positive urine, comparing differences according to the method of urine testing: urine diagnosis by filtration is shown by the black ellipses, sedimentation by the blue triangles, and centrifugation by the red diamonds. Studies that did not indicate an egg detection method are shown by green squares. Corresponding ROC curves are shown in the same colors. Symbol size is proportional to study enrollment.
(TIF)

Table S1 **Included studies.** Research survey reports included in this meta-analysis.
(XLSX)

Table S2 **Excluded studies.** Recovered studies given full review but ultimately excluded from meta-analysis.
(XLSX)

Table S3 **Meta-regression for sources of heterogeneity-School age surveys.**
(DOCX)

Table S4 **Meta-regression for sources of heterogeneity-Community surveys.**
(DOCX)
