# Supplementary figures and images for: Meta-analysis of Urine Heme Dipstick Diagnosis of Schistosoma haematobium Infection, Including Low-Prevalence and Previously-Treated Populations
Source: PLoS Negl Trop Dis. 2013 Sep 12;7(9):e2431. doi: 10.1371/journal.pntd.0002431 (PMC3772022; doi:10.1371/journal.pntd.0002431)

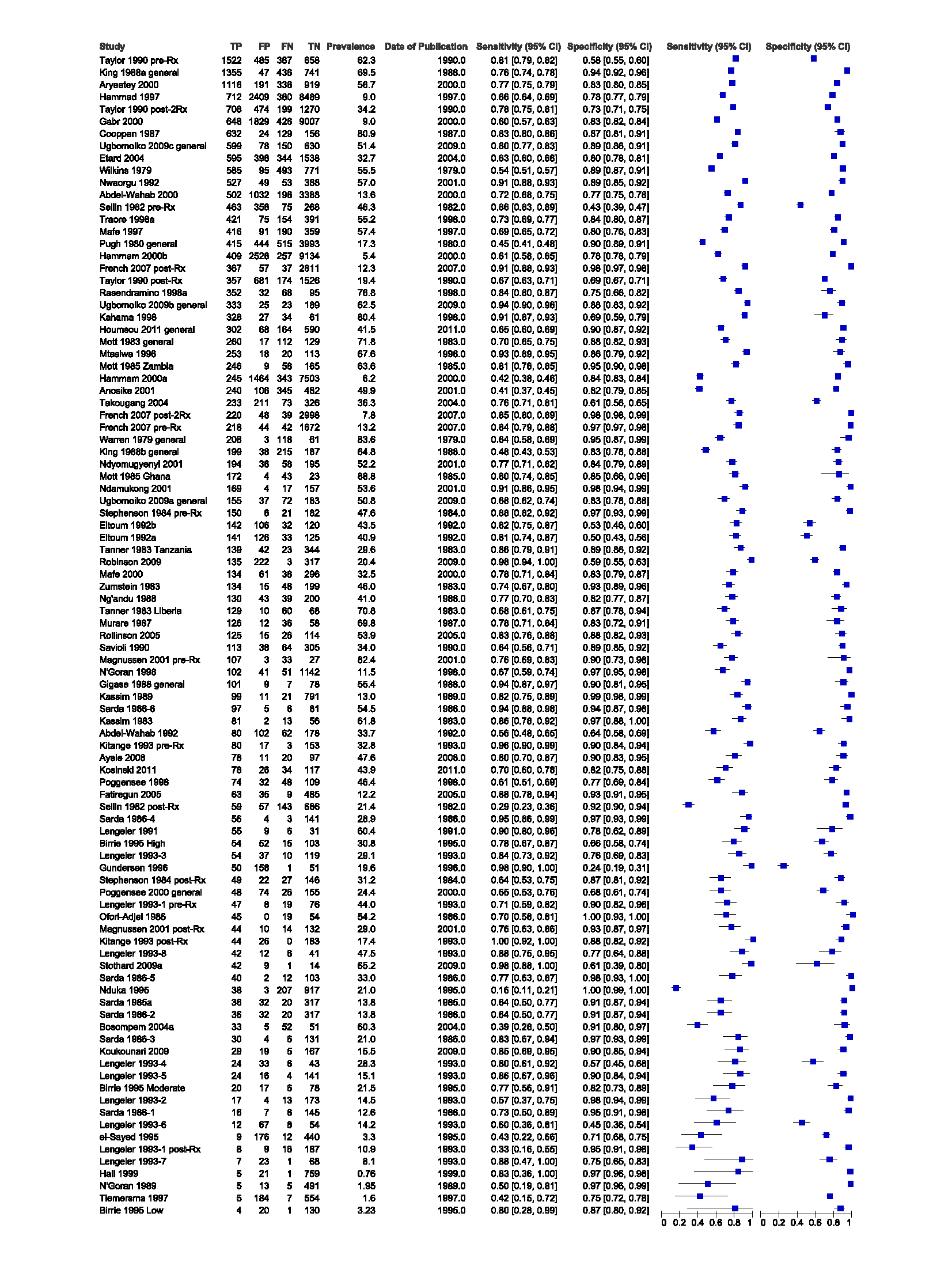

Supplement: Figure S1 — Forest plot of dipstick sensitivity and specificity according to size of the study population. (TIFF) [file pntd.0002431.s002.tif]

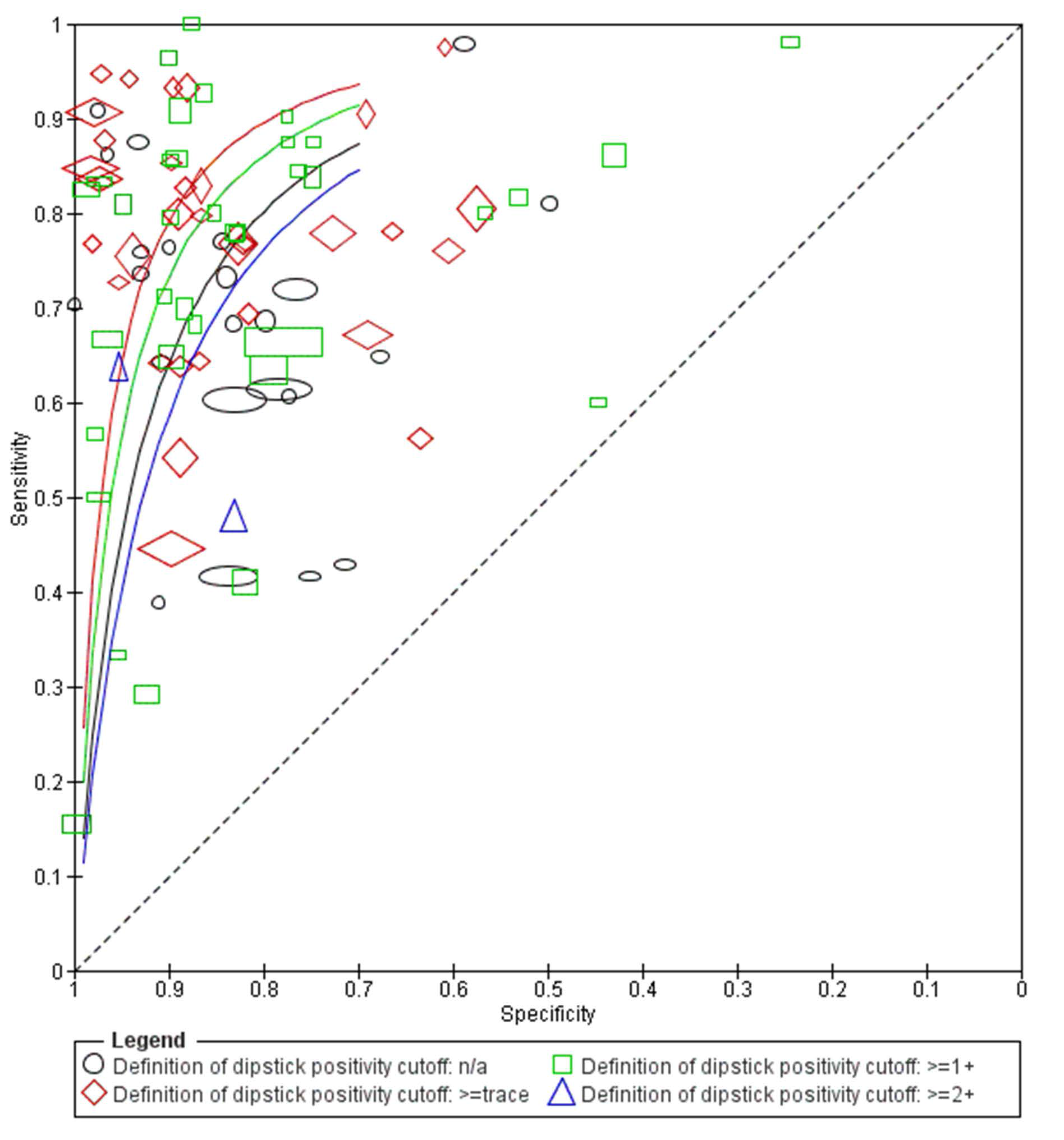

Supplement: Figure S2 — Diagnostic performance according to cutoff standards for the semi-quantitative heme reaction on a dipstick pad. Exploratory Moses-Shapiro-Littenberg SROC analysis curves indicate the relative performance of dipsticks for diagnosis of egg-positive urine, comparing studies using > = trace (red diamonds) vs. > = 1+ readout (green squares) vs. > = 2+ readout (blue triangles) for their cutoff criterion to define ‘hematuria positive’. Those studies that did not indicate a cutoff are shown by the black ellipses. Corresponding ROC curves are shown in the same colors. Symbol size is proportional to study enrollment. (TIF) [file pntd.0002431.s003.tif]

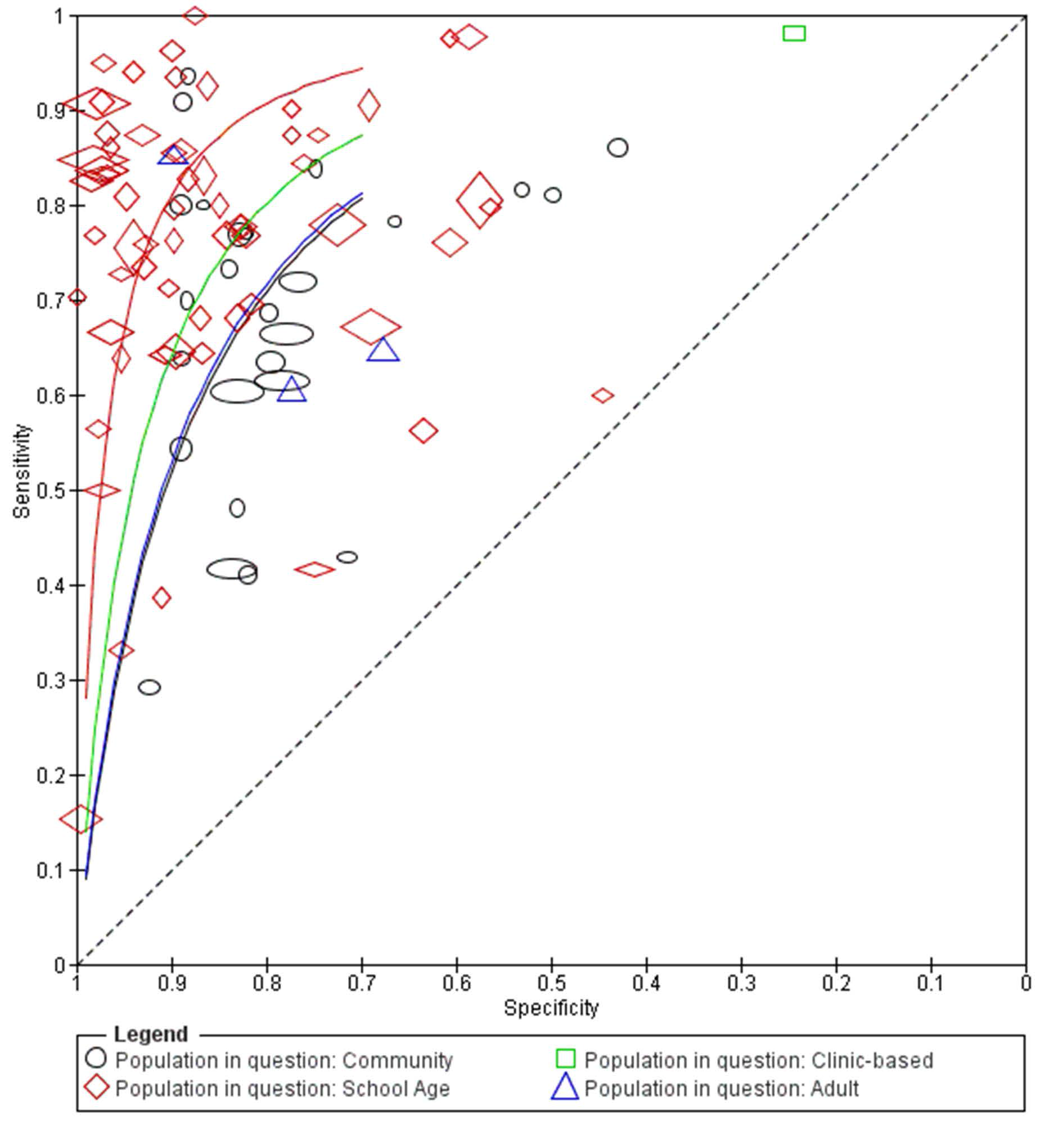

Supplement: Figure S3 — Diagnostic performance according to the age groups included in a dipstick diagnostic study. Exploratory Moses-Shapiro-Littenberg SROC analysis curves indicating the relative performance of dipsticks for diagnosis of egg-positive urine, comparing studies performed on communities at large (black ellipses, black line), school age children only (red diamonds, top red line), adults only (blue triangles, blue line), or clinic-based adults (green-squares, middle green line). Symbol size is proportional to study enrollment. (TIF) [file pntd.0002431.s004.tif]

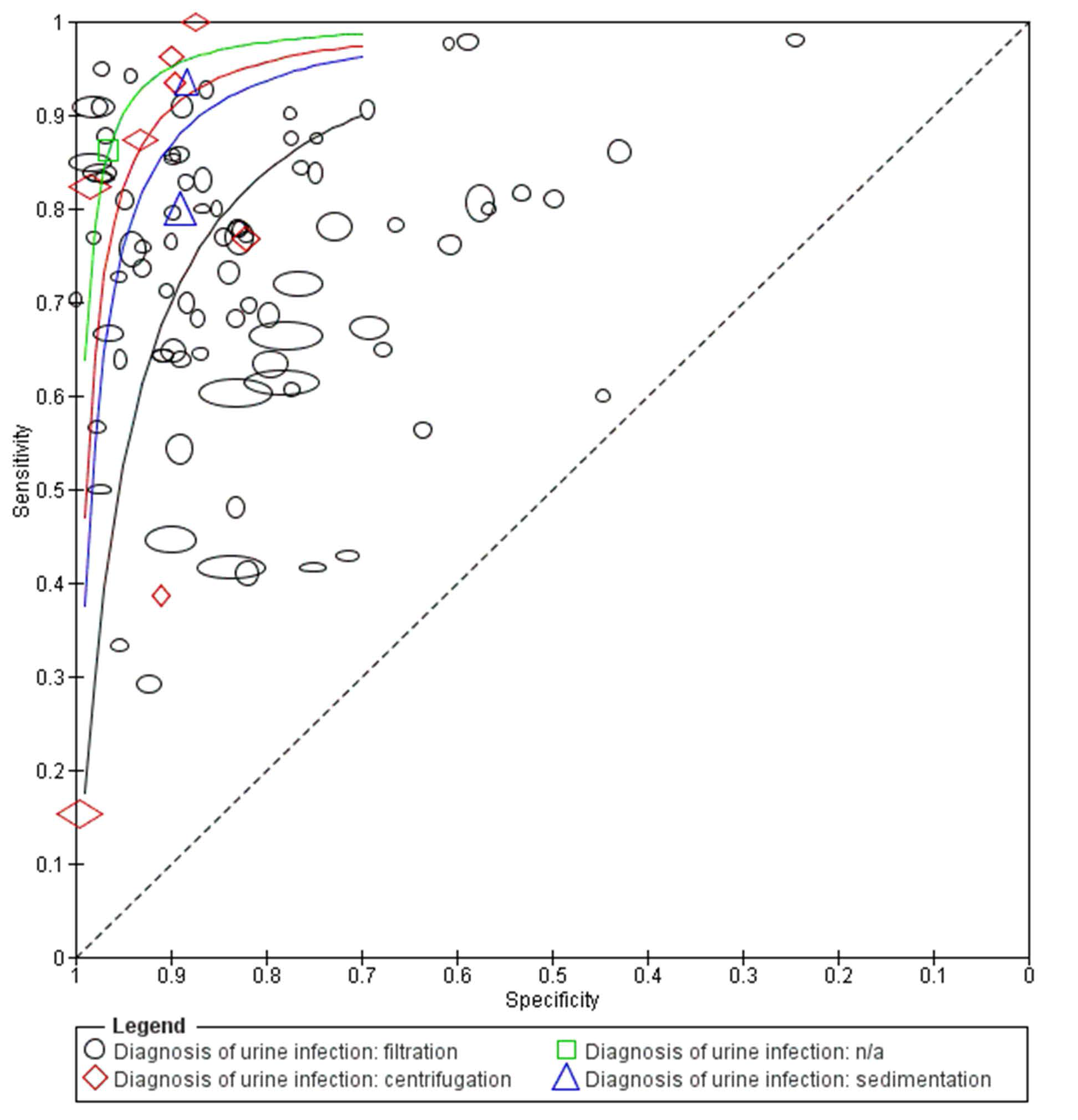

Supplement: Figure S4 — Dipstick diagnostic performance for detection of egg-positive urine according to the method of egg detection. Exploratory Moses-Shapiro-Littenberg SROC analysis curves indicating the relative performance of dipsticks for diagnosis of egg-positive urine, comparing differences according to the method of urine testing: urine diagnosis by filtration is shown by the black ellipses, sedimentation by the blue triangles, and centrifugation by the red diamonds. Studies that did not indicate an egg detection method are shown by green squares. Corresponding ROC curves are shown in the same colors. Symbol size is proportional to study enrollment. (TIF) [file pntd.0002431.s005.tif]
