# Supplementary material for: Meta-analysis of Urine Heme Dipstick Diagnosis of Schistosoma haematobium Infection, Including Low-Prevalence and Previously-Treated Populations
Source: PLoS Negl Trop Dis. 2013 Sep 12;7(9):e2431. doi: 10.1371/journal.pntd.0002431 (PMC3772022; doi:10.1371/journal.pntd.0002431)
Supplement: Table S3 — Meta-regression for sources of heterogeneity-School age surveys. (DOCX) [file pntd.0002431.s008.docx]

| **Covariate** | **Singly-adjusted** | | | **Multiply-adjusted^a^** | | |
| --- | --- | --- | --- | --- | --- | --- |
|  | **RDOR** | **95% CI** | **P value** | **RDOR** | **95% CI** | **P value** |
| Low Prevalence | 1.31 | 0.58, 2.94 | 0.51 |  |  |  |
| Post-Rx | 0.57 | 0.21, 1.59 | 0.28 |  |  |  |
| Brand 1 | --^b^ | -- | -- |  |  |  |
| Brand 2 | 0.79 | 0.26, 2.44 | 0.68 |  |  |  |
| Brand 3 | -- | -- | -- |  |  |  |
| Brand 4 | -- | -- | -- |  |  |  |
| Brand 5 | **0.41** | **0.17, 0.98** | **0.045** | **0.36** | **0.17, 0.77** | **0.009** |
| Brand 6 | 0.29 | 0.02, 4.40 | 0.37 |  |  |  |
| Brand 7 | 1.71 | 0.76, 3.85 | 0.19 |  |  |  |
| Brand 8 | 0.32 | 0.09, 1.10 | 0.069 | **0.27** | **0.09, 0.75** | **0.013** |
| Brand 9 | 2.01 | 0.77, 5.24 | 0.152 |  |  |  |
| Brand 10 | 1.22 | 0.16, 9.12 | 0.84 |  |  |  |
| Brand 11 | -- | -- | -- |  |  |  |
| Brand 12 | 1.22 | 0.35, 4.3 | 0.75 |  |  |  |
| Egg detection | 0.45 | 0.18, 1.10 | 0.079 | **0.45** | **0.21, 0.97** | **0.041** |
| Central Africa | 1.85 | 0.36, 9.62 | 0.46 |  |  |  |
| East Africa | **2.23** | **1.13, 4.43** | **0.022** |  |  |  |
| North Africa | **0.06** | **0.01, 0.37** | **0.003** | **0.07** | **0.01, 0.35** | **0.002** |
| South Africa | **0.35** | **0.13, 0.93** | **0.036** | **0.24** | **0.10, 0.56** | **0.001** |
| West Africa | 0.97 | 0.39, 2.43 | 0.95 |  |  |  |
| Study Era | 0.86 | 0.55, 1.34 | 0.50 |  |  |  |
| Dipstick Threshold | 0.96 | 0.61, 1.51 | 0.86 |  |  |  |

**Supplemental Table S3: Exploration for sources of heterogeneity—Relative covariate effects (singly- or multiply-adjusted) on diagnostic Odds Ratios for dipstick diagnosis of egg-positive urine among studies of school-age children (N=64)**

^a^A best fit reduced multivariable adjusted model was selected by stepwise backward removal of non-significant cofactors from a full model containing all covariates. The final working model presented here was selected when all variables in the model were either statistically significant or biologically plausible and marginally significant.

^b^ The dashes indicate insufficient data in this category for an estimate.
