# Supplementary material for: Meta-analysis of Urine Heme Dipstick Diagnosis of Schistosoma haematobium Infection, Including Low-Prevalence and Previously-Treated Populations
Source: PLoS Negl Trop Dis. 2013 Sep 12;7(9):e2431. doi: 10.1371/journal.pntd.0002431 (PMC3772022; doi:10.1371/journal.pntd.0002431)
Supplement: Table S4 — Meta-regression for sources of heterogeneity-Community surveys. (DOCX) [file pntd.0002431.s009.docx]

| **Covariate** | **Singly-adjusted** | | | **Multiply-adjusted^a^** | | |
| --- | --- | --- | --- | --- | --- | --- |
|  | **RDOR** | **95% CI** | **P value** | **RDOR** | **95% CI** | **P value** |
| Low Prevalence | 0.89 | 0.41, 1.91 | 0.75 |  |  |  |
| Post-Rx | 0.73 | 0.26, 2.04 | 0.53 |  |  |  |
| Brand 1 | 0.59 | 0.25, 1.42 | 0.23 |  |  |  |
| Brand 2 | 0.77 | 0.19, 3.02 | 0.69 |  |  |  |
| Brand 3 | --^b^ | -- | -- |  |  |  |
| Brand 4 | -- | -- | -- |  |  |  |
| Brand 5 | 1.94 | 0.86, 4.37 | 0.11 |  |  |  |
| Brand 6 | 0.70 | 0.11, 4.28 | 0.69 | **0.09** | **0.03, 0.28** | **< 0.001** |
| Brand 7 | 1.62 | 0.27, 9.90 | 0.59 | **0.31** | **0.11, 0.91** | **0.035** |
| Brand 8 | 0.35 | 0.06, 2.17 | 0.25 | **0.04** | **0.01, 0.14** | **< 0.001** |
| Brand 9 | 1.22 | 0.43, 3.42 | 0.70 |  |  |  |
| Brand 10 | 2.00 | 0.30, 13.2 | 0.46 |  |  |  |
| Brand 11 | 0.70 | 0.18, 2.74 | 0.59 |  |  |  |
| Brand 12 | -- | -- | -- |  |  |  |
| Egg detection | **6.40** | **2.09, 19.6** | **0.002** |  |  |  |
| Central Africa | -- | -- | -- |  |  |  |
| East Africa | 0.52 | 0.25, 1.10 | 0.086 | **0.40** | **0.25, 0.66** | **0.001** |
| North Africa | 0.60 | 0.25, 1.44 | 0.242 | **0.19** | **0.10, 0.35** | **< 0.001** |
| South Africa | 1.39 | 0.21, 9.22 | 0.72 |  |  |  |
| West Africa | **2.16** | **1.16, 4.05** | **0.017** |  |  |  |
| Study Era | 1.30 | 0.84, 2.01 | 0.23 | **2.83** | **1.95, 4.11** | **< 0.001** |
| Dipstick Threshold | 1.08 | 0.73, 1.58 | 0.70 | **1.47** | **1.12, 1.93** | **0.008** |

**Supplemental Table S4: Exploration for sources of heterogeneity—Relative covariate effects (singly- or multiply-adjusted) on diagnostic Odds Ratios for dipstick diagnosis of egg-positive urine among community-wide survey studies (N=30)**

^a^A best fit reduced multivariable adjusted model was selected by stepwise backward removal of non-significant cofactors from a full model containing all covariates. The final working model presented here was selected when all variables in the model were either statistically significant or biologically plausible and marginally significant.

^b^ The dashes indicate insufficient data in this category for an estimate.
